# Flocculation characteristics of a bioflocculant produced by the actinomycete *Streptomyces* sp. hsn06 on microalgae biomass

**DOI:** 10.1186/s12896-018-0471-9

**Published:** 2018-09-21

**Authors:** Yi Li, Yanting Xu, Ruixue Song, Congqi Tian, Lei Liu, Tianling Zheng, Hailei Wang

**Affiliations:** 1 College of Life Sciences, Henan Normal University, Xinxiang, 453007 China; 2 State Key Laboratory of Marine Environmental Science, School of Life Sciences, Xiamen University, Xiamen, 361005 China; 3 Xinxiang, China

**Keywords:** *Streptomyces* sp. hsn06, *Chlorella vulgaris*, Bioflocculant, Flocculation activity, Harvest of microalgal biomass

## Abstract

**Background:**

Microbial flocculation is a good choice for harvest of microalgae biomass, which has gained extensive attention. There have been carried out massive studies in bacterial flocculation, many bacterial strains with flocculation activity were isolated and different types of bioflocculants were produced. However, harvest of algal biomass by bioflocculants which produced from actinomycete are deficiency. In this study, the bioflocculant from an actinomycete *Streptomyces* sp. hsn06 could be used to harvest *Chlorella vulgaris* biomass.

**Results:**

Consecutive treatment with 20 mg·L^− 1^ bioflocculant and 5 mM CaCl_2_ for 5 min showed the highest flocculating activity. The bioflocculant was a nonprotein substance with thermal stability and pH stability, which can be used in comprehensive applications. Chemical analysis of the bioflocculant indicated that it is a small molecule substance of moderate polarity with containing triple bond and cumulated double bonds. Algal temperature, pH, and metal ions showed great effects on the flocculation efficiency of the bioflocculant.

**Conclusions:**

The bioflocculant produced by *Streptomyces* sp. hsn06 possesses the potential to harvest algal biomass with high-efficiency.

**Electronic supplementary material:**

The online version of this article (10.1186/s12896-018-0471-9) contains supplementary material, which is available to authorized users.

## Background

Algal biofuels are one of the best options for alleviating energy crisis [1]. Meanwhile, Compared with traditional fossil fuels, algal biofuels are more environmentally friendly and safety, which have got more and more focus [2]. *Chlorella vulgaris* with high growth rate and biomass yield is a common algal biofuel producing species [3], and possess great potential and value to realize industrialization. However, large-scale production of *C. vulgaris* biomass is not yet implemented, this is mainly due to the limitation of biomass harvesting [4]. The small size of *C. vulgaris* cells promotes algal cells to maintain colloidal stability in liquid, especially in mass culture, it’ s hard to separate algal cells from massive culture medium [5]. Therefore, harvesting of *C. vulgaris* biomass has become predominate bottleneck for development of *C. vulgaris* biofuel.

Harvest of algal biomass is a significant challenge in the field of microalgal biomass production, and different harvesting strategies including mechanical, electrical, chemical and biological methods have been used to harvest algal biomass [6]. For mechanical methods, algal cells can be harvested by centrifugation or filtration, but these manipulations will consume large amounts of energy with a higher cost [7]. Electrical methods will cause pollution of algal biomass by introducing metal ions, and lower the quality of algal biomass and limit recycling of algal culture medium [8]. Chemical methods are harmful to environment and cause secondary pollution [9]. Therefore, to minimize the energy consumption of harvesting microalgae biomass, the inexpensive, safety and effective harvesting technology should be exploited.

Compared with the above methods, bioflocculants mainly including natural biological polymers or extracellular polymers (Additional file 1: Table S1) which produced by microorganism can harvest microalgal cells safety and efficiently with avoiding contamination of biomass [10]. There have been reported different types of bioflocculants can be performed to harvest algal biomass, mainly belonged to protein, polysaccharide and other extracellular polymers with positive charges [11]. So far, many studies of bioflocculants produced from bacteria have been carried out. In previous work, we also confirmed that extracellular bioflocculant from the bacterium *Shinella albus* xn-1 can be used to harvest *C. vulgaris*. However, harvest of algal biomass by bioflocculants which produced from actinomycete have not been extensively studied.

We previously reported an actinomycete *Streptomyces* sp. hsn06 with the ability to harvest *C. vulgaris* biomass through mycelial pellets with direct flocculating algal cells [12]. Through further study, we determine that *Streptomyces* sp. hsn06 can secrete bioflocculant to flocculate algal biomass indirectly. To further investigate the characteristic of bioflocculant which produced by strain hsn06, in our present study we conducted the detailed investigation on flocculation activity of bioflocculant, the properties of bioflocculant, effect of different flocculation conditions on flocculation activity, and formation of the flocs.

## Methods

### Algal cultures and actinomycete cultures

The algae *C. vulgaris* was supplied by Freshwater Algae Culture Collection, Institute of Hydrobiology, Wuhan, China. The *C. vulgaris* strain was cultivated in BG-11 medium [13] at 25 ± 1 °C under a 12:12 h light-dark cycle with a light intensity of 50 μmol photons m^− 2^ s^− 1^. Exponential phase axenic cultures were aliquoted for further experiments.

Actinomycete *Streptomyces* sp. hsn06 was cultured in flocculation medium (glucose, 10 g; NaCl, 24 g; NH_4_Cl, 1 g; MgSO_4_⋅7H_2_O, 0.5 g; yeast extract, 0.6 g; K_2_HPO_4_⋅3H_2_O, 6.5 g and KH_2_PO_4_, 2 g in 1 L distilled water) for 2 days at 28 °C with shaking at 120 rpm.

### Production and purification of bioflocculant

The bioflocculant was purified according to the previous method [14]. The supernatant was collected by centrifugation at 8000 rpm for 10 min at 4 °C, and bioflocculant was purified as follows: The supernatant was subsequently added to three volumes of cold ethanol, and then incubated at 4 °C for 24 h. The whole mixture was centrifugated at 8000 rpm for 10 min, and the resulting precipitate was collected and dissolved in deionized water. The supernatant was subsequently added to cold ethanol, and the mixture was then incubated at 4 °C for 24 h. The resulting precipitate was treated according to the above steps again, and all the supernatants were combined together, and then lyophilized to obtain purified bioflocculant FLC-hsn06.

### Flocculation activities of bioflocculants with different concentrations

The purified bioflocculant was added into sterile distilled water with the final concentration of 10 g·L^− 1^. Different concentrations of bioflocculants including 1, 3, 5, 10, 20, 30, 40, 50, and 100 mg·L^− 1^ were added into *C. vulgaris* cultures to investigate the flocculation activities, respectively. The normal growth algae were set as control group. Final concentration of 5 mM CaCl_2_ was added in each treatment groups and control, followed by mixing at 120 rpm for 5 min at room temperature, and then the mixing was stopped to let the mixture settle at 25 °C. After the algal cells were aggregated, an aliquot of the culture in treatment groups and control was pipetted from a height of two-thirds from the bottom for the evaluation of the flocculation effect using a spectrometer, which was measured at a wavelength of 680 nm. All the treatment groups and control group were carried out in triplicate.

The flocculation activity was calculated according to the following formula

1 $$ \mathrm{Flocculation}\kern0.17em \mathrm{activity}\;\left(\%\right)=\left(A-B\right)/A\times 100 $$

where A and B are the absorbency (OD) at 680 nm of the microalgal culture in control and treatment groups, respectively.

### Characteristics of bioflocculant FLC-hsn06

To determine the characteristics of bioflocculants, the flocculation activities of bioflocculants were investigated after treated under different temperatures, pH, and proteinase K treatment. 20 mg·L^− 1^ of bioflocculants were incubated at 40, 60, 80, 99 and 121 °C for 2 h, respectively. Then, the heated bioflocculants were added into algal culture after cooling to room temperature. Control group was normal growth algae, positive control was normal growth algae with adding untreated bioflocculants. The pH of the bioflocculant was adjusted to 3, 5, 7, 9 and 11 and maintained for 1 h, and then adjusted to the initial pH. The treated bioflocculants were added into algal cultures to determine flocculation efficiency. Control group was normal growth algae, positive control was normal growth algae with adding untreated bioflocculants. The bioflocculants were digested with 100 mg·L^− 1^ proteinase K in sterile BG11 at pH 7.4. Samples were incubated for 2 h at room temperature. The treated bioflocculants were then added into algal culture to test the flocculation activity, normal growth algae with adding same concentration of proteinase K was set as control, positive control was normal growth algae with adding untreated bioflocculants. Final concentration of 5 mM CaCl_2_ was added in each treatment groups and controls, followed by mixing at 120 rpm for 5 min. All the treatment groups and control groups were carried out in triplicate. The flocculation activity of each treatment groups was determined according to Eq. (1). The total sugar content of FLC-hsn06 was determined following the phenol-sulfuric acid colorimetric method using glucose as standard. The protein content of the bioflocculant FLC-hsn06 was measured using the Coomassie brilliant blue protein analysis kit (Nanjing Jiancheng Bioengineering Institute, China) with bovine serum albumin as the standard.

### Analysis of bioflocculant FLC-hsn06

To determine the polarity of bioflocculant FLC-hsn06, different organic solvents including n-butyl alcohol, ethyl acetate, trichloromethane, dichloromethane, and methylbenzene were used to extract the flocculation compound from bioflocculant FLC-hsn06. The bioflocculant was mixed with the organic solvent for 2 h. The mixture was stood for 30 min to collect the organic phase, and evaporated to dryness and weighed to obtain extracts. All extracts were dissolved in dimethyl sulfoxide (DMSO) to test the flocculation activities, and algal culture with adding same volume of DMSO was set as a negative control. The flocculation compound after extracted from bioflocculant FLC-hsn06 by dichloromethane was further investigated based on analysis of thin layer chromatography (TLC, GF254, pH: 6.2–6.8, Qingdao, China) using a dichloromethane: n-hexane: acetic acid = 50: 1: 0.5 as the spreading agent and by means of high performance liquid chromatography (HPLC) and, to ensure the purity of the flocculation compound, fitted with a SunFireTm C18 (4.6 × 250 mm, 5 μm) column using a methanol: 25% of KH_2_PO_4_ = 3:1 (*v*/v) mixture as the eluent at a flow rate of 1 mL·min^− 1^ with 480 nm as the detection wavelength.

### Functional groups of bioflocculant FLC-hsn06

The Fourier transform infrared (FITR) analysis was used to determine the chemical groups of flocculation compound, to further determine the function group during flocculation procedure, dialysis treatment on bioflocculant was performed firstly. The bioflocculant was loaded into dialysis bags with molecular intercept values of 1 kD and dialyzed in sterile distilled water for 2, and 4 h, respectively. The bioflocculant after dialysis were added into algal culture to test the flocculation activity. Normal growth algae were set as control, the positive control was normal growth algae with the addition of undialysis bioflocculant. And then the characteristic chemical groups of the bioflocculant FLC-hsn06 before and after dialysis were analyzed by the Fourier transform infrared (FITR) spectrometer (ThermoFisher NEXUS, America) in the frequency of 400–4000 cm^− 1^.

### Effects of different flocculation conditions on flocculation activity

The flocculation experiments were performed under different algal temperatures including 10, 20, 30, and 40 °C with bioflocculant FLC-hsn06 at a concentration of 20 mg⋅L^− 1^ and 5 mM CaCl_2_. The pH of algal culture was adjusted to 6, 7, 8, 9, 10, 11, and 12 before the experiment, and then bioflocculant FLC-hsn06 at a concentration of 20 mg⋅L^− 1^ were added into different pH treatment groups with 5 mM CaCl_2_, the control groups were the algal cells with 5 mM CaCl_2_ and same pH values. Different concentrations (0.1, 1, 3 and 5 mM) of metal ions with various electrical charges including CaCl_2_, FeCl_3_, CuCl_2_, MgCl_2_, NaCl, and KCl were tested as cationic coagulants for the flocculation of *C. vulgaris* culture. Control was prepared using the same procedure with the addition of same metal ions with same concentrations.

### Flocculation process of bioflocculant FLC-hsn06

The bioflocculant FLC-hsn06 at a concentration of 20 mg⋅L^− 1^ was added into *C. vulgaris* culture with 5 mM CaCl_2_, followed by mixing quickly, and then 10 μL of the algal cells at the bottom after flocculation treatment for 1, 2, 3, 4, and 5 min was used to investigated the flocculation procedure based on the microscope (Olympus BX41, Chiyoda-ku, Tokyo, Japan).

### Statistics

All data were presented as mean ± standard error and were evaluated using one-way analysis of variance followed by the least significant difference test, with *p* < 0.01 and *p* < 0.05 (Origin 8.5 for Windows).

## Results

### Flocculation activity of bioflocculants on algal cells

**Fig. 1:**
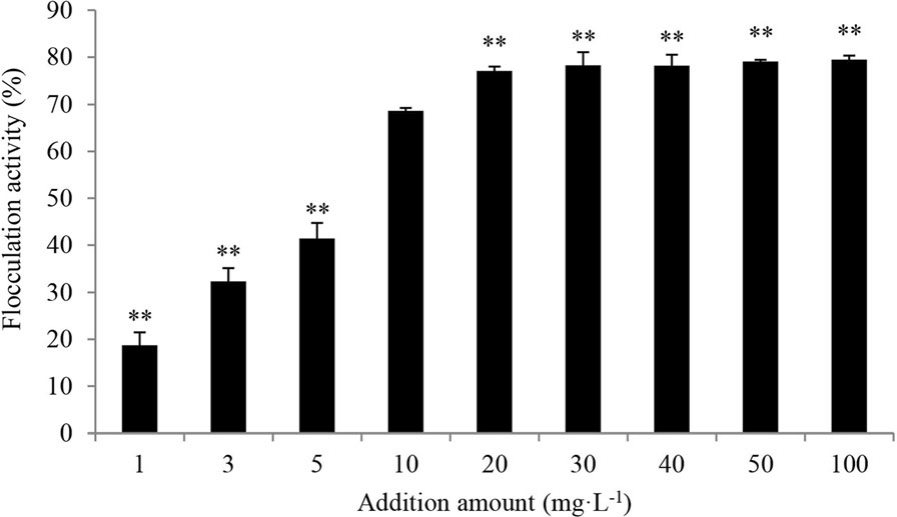
Flocculation activity of bioflocculant at concentrations of 1, 3, 5, 10, 20, 30, 40, 50, and 100 mg·L^− 1^ on *C. vulgaris*. All error bars indicate the SE of the three biological replicates. **Represents a statistically significant difference of *p* < 0.01 compared with flocculation activity of bioflocculant with the concentration of 10 mg·L^− 1^

To investigate the flocculation effect of different concentrations of bioflocculant on *C. vulgaris* cells, different concentrations of bioflocculant including 1, 3, 5, 10, 20, 30, 40, 50, and 100 mg·L^− 1^ were added into *C. vulgaris* cultures with adding final concentration of 5 mM CaCl_2_. As shown in Fig. 1, bioflocculant FLC-hsn06 showed obvious flocculation activity on algal cells, and flocculation rate increased with the increase of bioflocculant concentration. The flocculation effect was relatively low in the treatment groups with the 1, 3, and 5 mg·L^− 1^ concentrations of bioflocculant, which were only approximately 27.2, 47.1, and 60.3% of the treatment group with concentration of 10 mg·L^− 1^, respectively. The increase of the bioflocculant concentration can raise flocculation activity, the flocculation rate reached to 68.7% when the concentration of bioflocculant was 10 mg·L^− 1^. However, the flocculation efficiency achieved stability when concentrations of bioflocculant exceeded 10 mg·L^− 1^. The flocculation activities in 20, 30, 40, 50, and 100 mg·L^− 1^ treatment groups significantly (*p* < 0.01) higher than that in 10 mg·L^− 1^, and showed no obvious difference between high concentrations of bioflocculant (> 20 mg·L^− 1^). 20 mg·L^− 1^ of bioflocculant showed high flocculation activity as well as higher concentrations of bioflocculant, therefore, the concentration of 20 mg·L^− 1^ was the most suitable addition amount for bioflocculant to harvest algal cells.

### Characteristics of bioflocculant FLC-hsn06

**Fig. 2:**
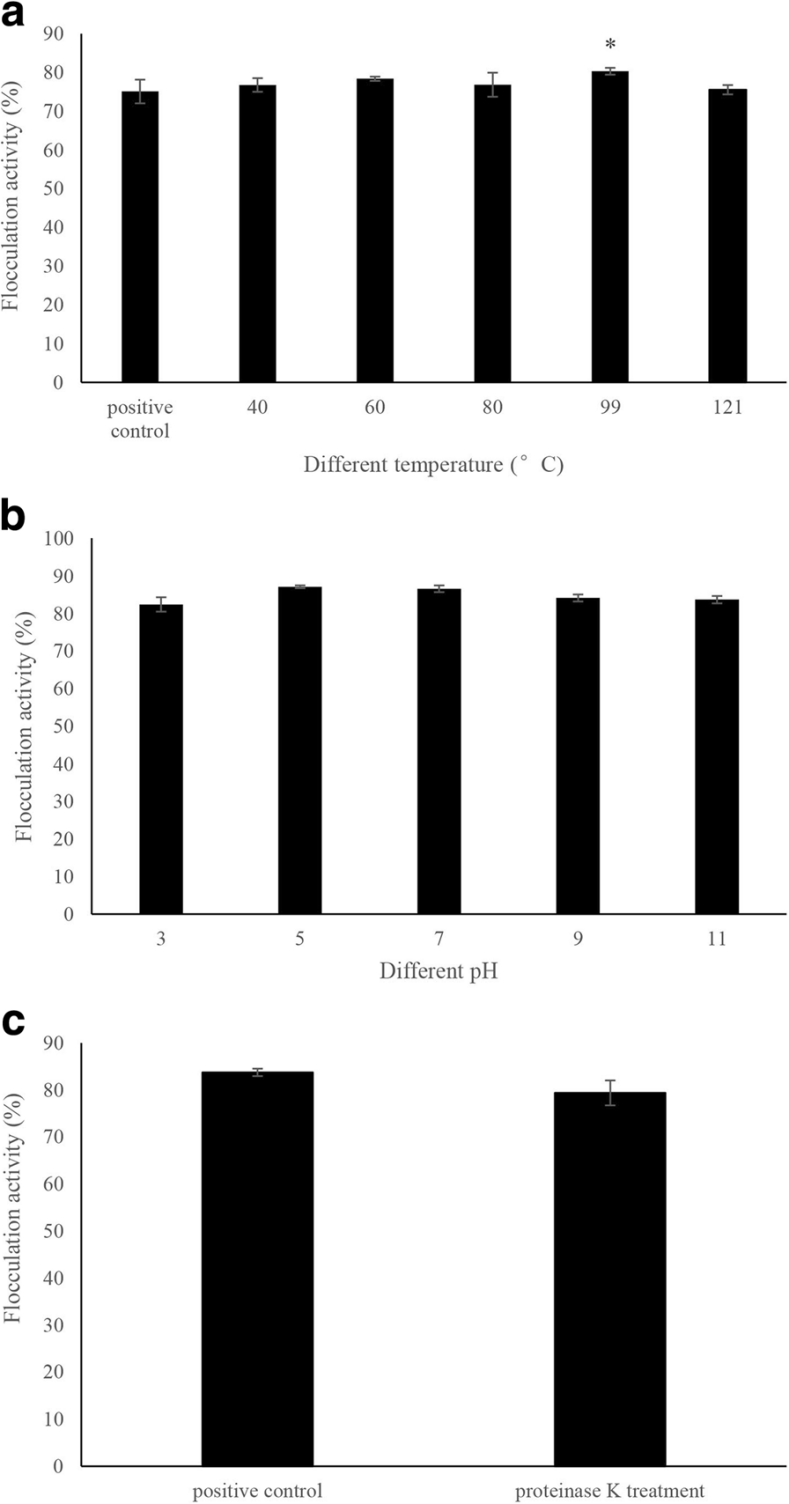
Effect of different temperatures (**a**), pH (**b**), and proteinase K digestion (**c**) on flocculation activity of the bioflocculant. All error bars indicate the SE of the three biological replicates. *represents a statistically significant difference of *p* < 0.05 compared with positive control

To determine the characteristics of bioflocculant FLC-hsn06, different treatments have been implemented on the bioflocculant. As shown in Fig. 2a, flocculation activity of bioflocculant FLC-hsn06 on algal cells after treated under different temperatures showed no difference compared to positive control. The bioflocculant FLC-hsn06 after heated under 99 °C even exhibited higher (*p* < 0.05) flocculation efficiency than that in positive control, which implied that bioflocculant FLC-hsn06 shows flocculation activity with thermostability. As shown in Fig. 2b, there showed no obvious differences between different treatment groups with adding bioflocculants after treated by each pH values, flocculation activities in all the treatment groups reached more than 80%. As shown in Fig. 2c, the flocculation activity of the bioflocculant after treated by proteinase K still did not show any obvious difference compared to positive control, and exhibited high flocculation effect on algal cells.

### Chemical properties of bioflocculant FLC-hsn06

**Fig. 3:**
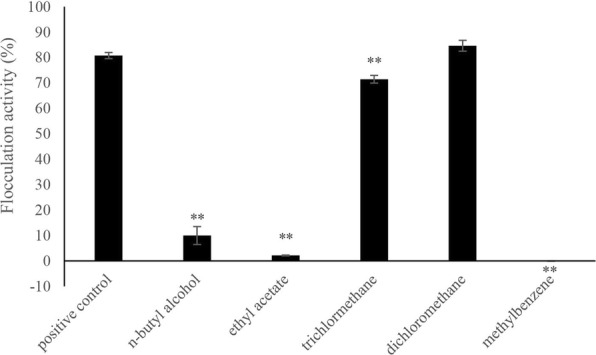
Flocculation activity of different organic solvents extracts from the bioflocculant. All error bars indicate the SE of the three biological replicates. **represents a statistically significant difference of *p* < 0.01 compared with positive control

Five kinds of organic solvents including n-butyl alcohol, ethyl acetate, trichloromethane, dichloromethane, and methylbenzene which possessed different polarity were used to extract active compound from bioflocculant FLC-hsn06. As shown in Fig. 3, the three organic solvents including n-butyl alcohol, ethyl acetate, and methylbenzene could not extract the active component from bioflocculant, the flocculation activities were significantly (*p* < 0.01) lower than positive control. The trichloromethane extract and dichloromethane extract showed high flocculation activity on algal cells. Among of them, the flocculation rate of trichloromethane extract was obviously (*p* < 0.01) lower than that in positive control, which was 88.5% of the positive control. However, there showed no difference between positive control and dichloromethane extract, suggesting the polarity of active compound in bioflocculant FLC-hsn06 is close to dichloromethane, which belongs to moderate polarity substance. According to the results, dichloromethane was confirmed as the suitable organic solvent to extract active compound, and the dichloromethane extract was analysed through thin layer chromatography (TLC) and high performance liquid chromatography (HPLC). As shown in Additional file 2: Figure S2, the TLC mainly contained one obvious band, which indicated the dichloromethane extract included a few substances, and this band maybe represented active compound in bioflocculant FLC-hsn06. However, the HPLC analysis showed three separate peaks, which represented three different substances. Among of the three peaks, the third peak was significantly higher than the other peaks, suggesting this peak which represented active compound maybe plays an important role in flocculation activity.

### Functional groups of bioflocculant FLC-hsn06

**Fig. 4:**
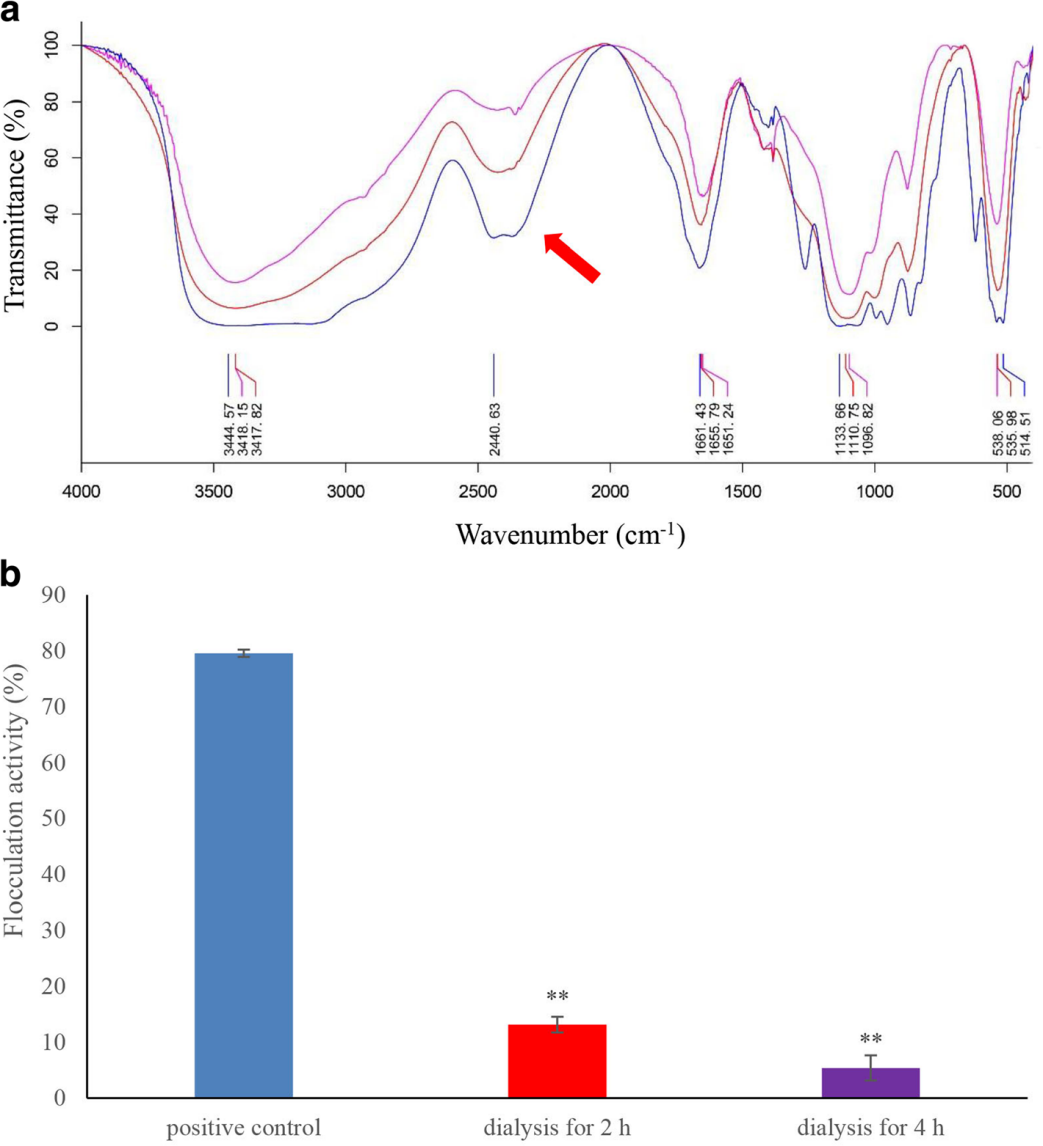
Fourier transform-infrared spectrograms (**a**) and flocculation activity (**b**) of the bioflocculant before and after dialysis. All error bars indicate the SE of the three biological replicates. **represents a statistically significant difference of *p* < 0.01 compared with positive control

The functional groups of bioflocculant FLC-hsn06 before and after dialysis were analyzed using FTIR spectroscopy; the spectra at 400–4000 cm^− 1^ are reproduced in Fig. 4a. The results showed a strong absorption peak were found in the bioflocculant FLC- hsn06 before dialysis at 3445 cm^− 1^, 2441 cm^− 1^, an asymmetrical stretching peak observed at 1661 cm^− 1^, absorption peak at 1134 cm^− 1^. However, the absorption peaks showed obvious changes in the bioflocculant FLC-hsn06 after dialysis treatment for 2 h and 4 h. Several strong absorption peaks were showed up at 3418, 1656, 1111, and 536 cm^− 1^ in the bioflocculant FLC-hsn06 after dialysis for 2 h, and the same absorption peaks were found in 3418, 1651, 1097, and 538 cm^− 1^ in the bioflocculant FLC-hsn06 dialysis for 4 h. Compared the absorption peaks of bioflocculant FLC-hsn06 before and after dialysis, there showed no difference between bioflocculant FLC-hsn06 after dialysis for different times, which contained four absorption peaks after dialysis. However, the absorption peaks of bioflocculant FLC-hsn06 before and after dialysis showed significantly distinctions, one absorption peak (2441 cm^− 1^) disappeared after dialysis. To determine the effect of dialysis on the bioflocculant, the flocculation activity of the bioflocculant before and after dialysis was investigated. As shown in Fig. 4b, flocculation activities of bioflocculant after dialysis for 2 h and 4 h were significantly (*p* < 0.01) lower than that in bioflocculant before dialysis, which were only 16.5% and 6.8% of positive control, respectively. After the dialysis treatment, there showed no flocculation activity inside the dialysis bag, which confirmed that active compound can pass through dialysis bag with molecular intercept values of 1 kD, and that the molecular weight of the bioflocculant is less than 1 kD. According to the FTIR analysis and dialysis treatment, the bioflocculant lost flocculation activity after dialysis as well as the disappear of absorption peak of 2441 cm^− 1^, suggesting that active compound in the bioflocculant causes absorption peak of 2441 cm^− 1^.

### Effects of different flocculation conditions on flocculation activity

**Fig. 5:**
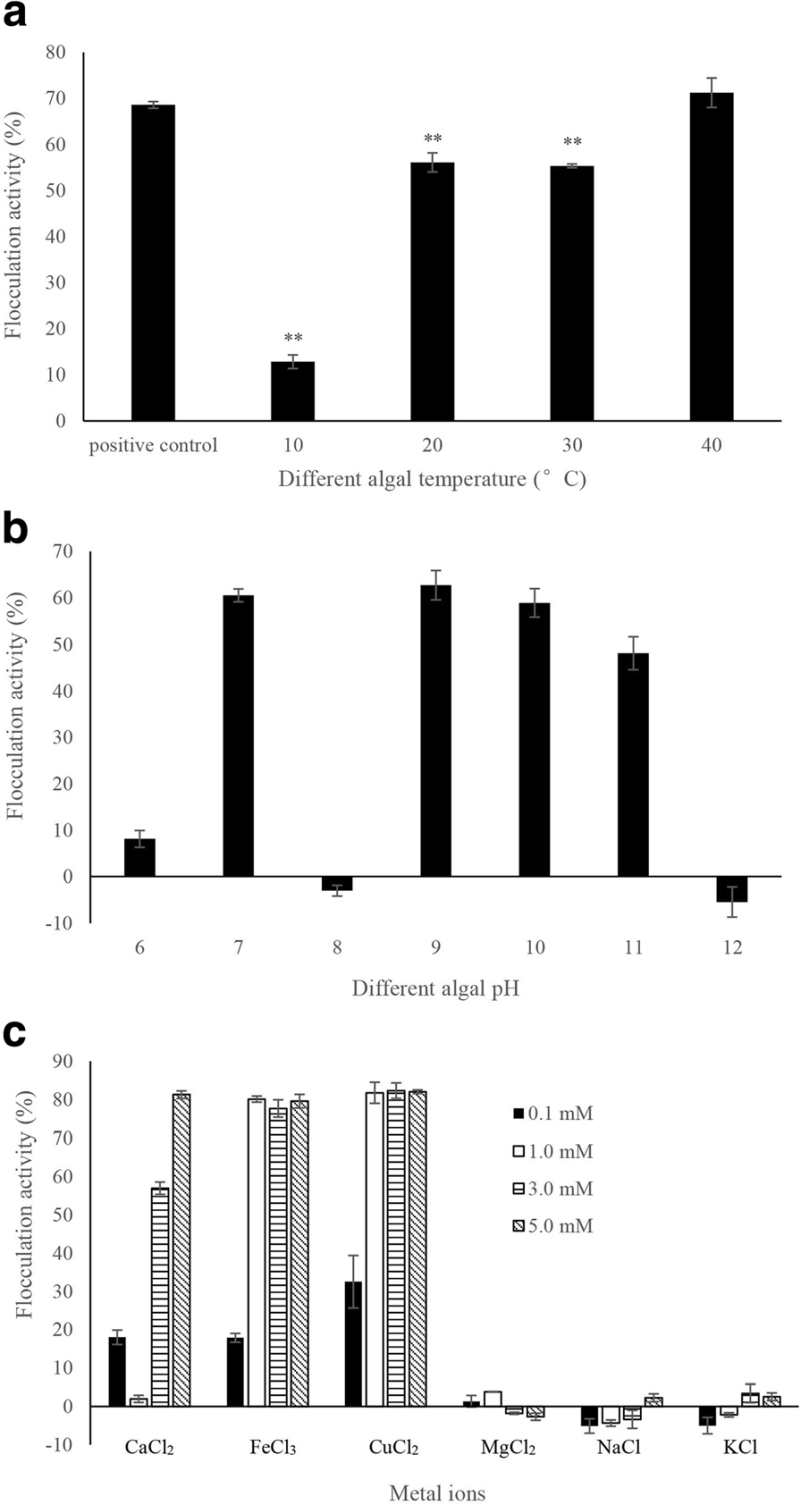
The effect of flocculation conditions including different algal temperature (**a**), algal pH values (**b**) and metal ions (**c**) on flocculation activity of bioflocculant. All error bars indicate the SE of the three biological replicates. **represents a statistically significant difference of *p* < 0.01 compared with positive control

As shown in Fig. 5a, flocculation activity of the bioflocculant showed great differences under different algal temperatures. Flocculation activities of bioflocculant under different algal temperatures including 10, 20, and 30 °C were significantly (*p* < 0.01) lower than that in positive control, which were 18.6, 81.9, and 80.8% of positive control, respectively. However, the bioflocculant could exhibit high flocculation efficiency under 40 °C of algal temperature, compared to positive control.

To investigate the effect of algal initial pH on flocculation activity, the flocculation experiments under different algal pH values including 6, 7, 8, 9, 10, 11, and 12 were performed. The effect of pH on flocculation activity of bioflocculant FLC-hsn06 is shown in Fig. 5b. The flocculation activity was not stable under different algal pH, the treatment groups with pH of 6, 8 and 12 almost lost flocculation activities, and the flocculation activity was significantly lower than other treatment groups. Basic pH values (9–11) induced highest flocculation compared to other pH values, as well as pH value of 7.

To determine the effect of metal ions on flocculation activity of the bioflocculant, the flocculation efficiency when the metal ions including Ca^2+^, Fe^3+^, Cu^2+^, Mg^2+^, Na^+^, and k^+^ ions were added in the algal culture with bioflocculant FLC-hsn06 was investigated. As shown in Fig. 5c, flocculation activities of the bioflocculant with adding Mg^2+^, Na^+^, and k^+^ ions were significantly lower than the bioflocculant with adding Ca^2+^, Fe^3+^, Cu^2+^ ions as coagulants. The bioflocculant with adding 5 mM Ca^2+^, Fe^3+^, Cu^2+^ ions induced highest flocculation activity on algal cells, and Fe^3+^, Cu^2+^ ions could exhibit more efficient promotion effect on flocculation than Ca^2+^ ion under low concentrations. However, Fe^3+^ and Cu^2+^ ions with toxicity would pose a great threat on environment, therefore, Ca^2+^ ion could be selected as the most suitable coagulant to improve flocculation activity of the bioflocculant.

### Floc formation procedure

**Fig. 6:**
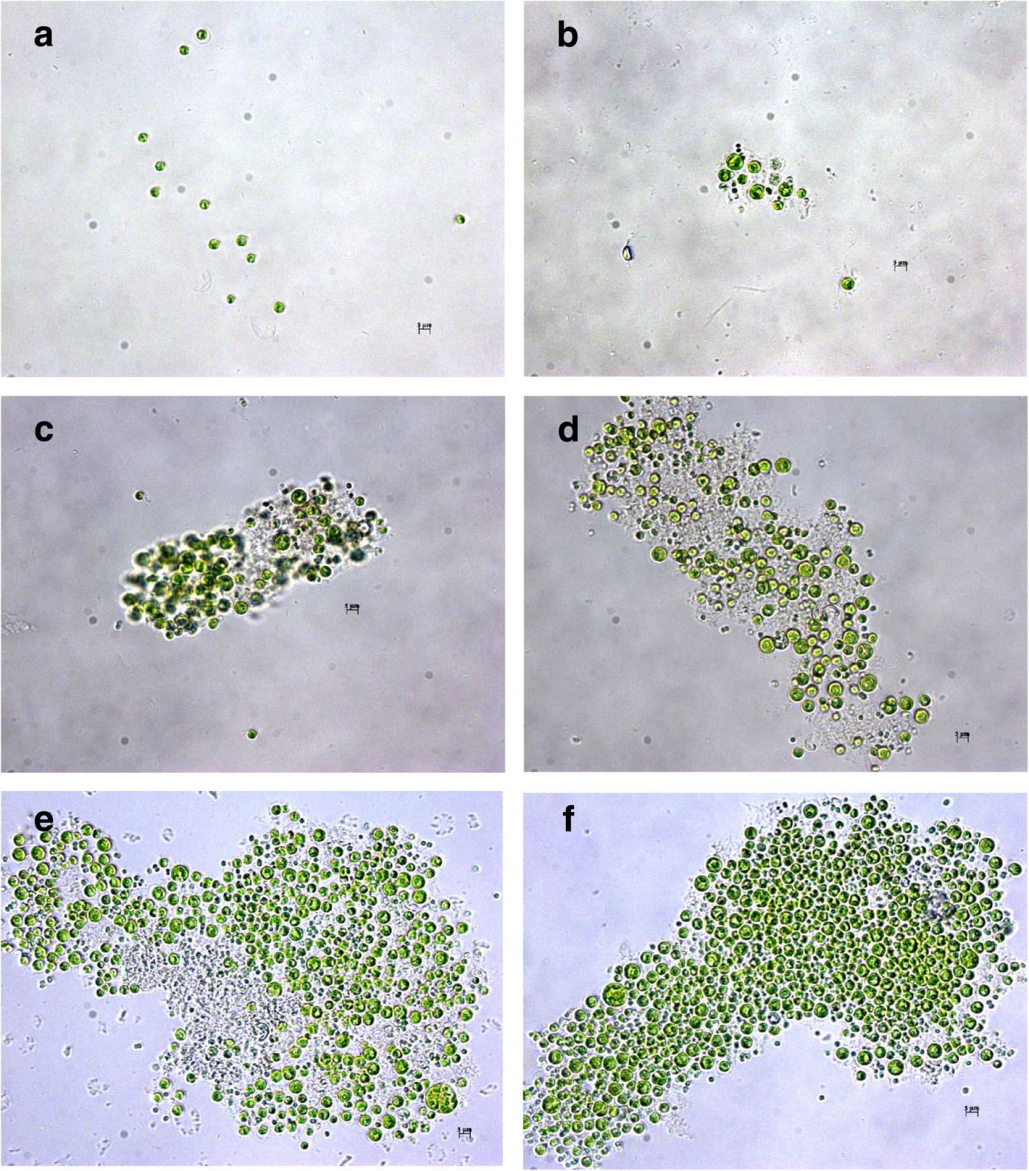
Flocculation process of *C. vulgaris* cells under the flocculation activity of bioflocculant for 0 (**a**), 1 (**b**), 2 (**c**), 3 (**d**), 4 (**e**) and 5 min (**f**) treatment time

Many conditions including different algal temperatures, pH will influence the formation of floc during the flocculation procedure under the flocculation activity of the bioflocculant (Fig. 5a, b). Floc formation is crucial for the bioflocculant to harvest algal biomass, therefore, the floc formation procedure was observed based on the microscopic observation. The algal flocculation procedure under the flocculation activity of the bioflocculant is shown in Fig. 6. The algal cells distributed uniformly in the liquid medium before adding the bioflocculant, and the single algal cell could be found clearly without existence of the floc (Fig. 6a). When the flocculation time was 1 min, algal cells began to gather together and small volume of floc could be observed in the field. With the increase of processing time, a large number of algal cells were gathered together to form larger volume of floc, and the volume of floc was getting bigger and bigger (Fig. 6b-e). When the flocculation time was 5 min, the flocculation activity reached the highest level (Fig. 1), meanwhile, the volume of floc reached maximum. The bioflocculant could neutralize the surface negative charges of algal cells to promote these cells to attract together, and then the heavy floc was formed as well as harvesting of algal cells.

## Discussion

Harvest of microalgal biomass by microorganism is a good choice for the development of industrialization of microalgae. More and more bioflocculants were isolated and performed to harvest microalgal biomass. In this study, from an actinomycete *Streptomyces* sp. hsn06 were investigated to harvest *C. vulgaris* biomass.

The flocculation activity of bioflocculants on *C. vulgaris* biomass was determined that the concentration of 20 mg·L^− 1^ was the most suitable addition amount for bioflocculant to harvest algal cells. In the previous research, we extracted bioflocculant FLC-xn-1 from the bacterium *Shinella albus* xn-1, the results showed that bioflocculant FLC-xn-1 exhibited high flocculation activity when the concentration of bioflocculant exceeded 30 mg·L^− 1^, and flocculation activity in 20 mg·L^− 1^ treatment group kept low level [14] Compared to bioflocculant FLC-xn-1, the bioflocculant FLC-hsn06 in this study possessed higher flocculation activity. Lei et al. have reported bioflocculant from *Cobetia marina* L03 could be used to effective harvest *C. vulgaris* via flocculation-flotation [15], which showed similar effects with bioflocculant FLC-hsn06. Among of them, 10 mg·L^− 1^ of bioflocculant resulted in a flotation efficiency of 62.5%, which was further increased to 92.7% when the dosage was increased to 20 mg·L^− 1^. Compared to these reported bioflocculant, the bioflocculant FLC-hsn06 exhibited flocculation activity on algal cells with high efficiency, which promise it to apply in harvesting algal biomass with low cost.

Most reported bioflocculants belonged to proteins, polysaccharides, or other extracellular polymeric substances [16, 17], among of them, proteins always lost bioactivity under high temperature with thermal instability. Li et al. investigated the flocculation activity of mycelial pellets of the actinomycete *Streptomyces* sp. hsn06 after treated under different temperatures on *C. vulgaris* cells, the results showed that the flocculation activities of mycelial pellets after treated under different temperatures were significantly (*p* < 0.01) decclined compared to control [12], which indicated that proteins maybe involve in flocculation procedure and contribute to flocculation activity. Acid and alkali treatments can induce proteins denaturation [18]. Li et al. have reported flocculation activity of mycelial pellets of *Aspergillus niger* after treated by acid (pH 3) and alkali (pH 12) treatment [19], which showed that flocculation activities in acid and alkali treatment groups were significantly (*p* < 0.01) lower than that in positive control, suggesting proteins are major factors in the flocculation process. Lei et al. have reported that bioflocculant L03 was stable within a wide range of pH values, between 4 and 10 [15], suggesting proteins maybe not major factor to induce flocculation activity. Therefore, bioflocculant FLC-hsn06 possessed pH stability and proteins in the bioflocculant may not be involved in flocculation activity. To further determine whether proteins in bioflocculant FLC-hsn06 contribute to flocculation activity, the flocculation activity of the bioflocculant after treated by proteinase K was investigated. Proteinase K can exhibit a broad cleavage activity for different proteins to cut the aliphatic amino acids and carboxy terminal peptide of aromatic amino acids [20], which can induce protein digestion with high specificity.

Li et al. have confirmed flocculation activity of mycelial pellets incubated with proteinase K was significantly (*p* < 0.01) lower than that of the nondigested controls [19], indicating that proteinase K-cleavable surface proteins are major factors in the flocculation process. Therefore, proteins in bioflocculant did not play any flocculation effect to harvest algal cells. And the protein contents in bioflocculant FLC-hsn06 were so low, which accounted for only 0.43% of the total (Additional file 3: Figure S1). The determination of characteristics of bioflocculant FLC-hsn06 can confirm that bioflocculant FLC-hsn06 is a nonprotein substance with thermal stability and pH stability, which can promise the bioflocculant has the potential to be used in comprehensive applications.

The chemical groups of flocculation compound were investigated by the FTIR analysis. The FTIR spectroscopy confirmed a strong absorption peak were found in the bioflocculant FLC- hsn06 before dialysis at 3445 cm^− 1^,generated by the stretching vibration of -OH or -NH groups [21]. Band at 2441 cm^− 1^ represents stretching vibration region of triple bond and cumulated double bonds [22]. An asymmetrical stretching peak observed at 1661 cm^− 1^ was for carbonyl group stretching vibration [17], absorption peak at 1134 cm^− 1^ indicated asymmetrical stretching vibration of an ester linkage, generally as typical characteristics of all sugar derivatives [17]. The active compound in the bioflocculant FLC- hsn06 causes absorption peak of 2441 cm^− 1^, and absorption peak of 2441 cm^− 1^ represents stretching vibration region of triple bond and cumulated double bonds. Therefore, the small molecule substance with containing triple bond and cumulated double bonds in the bioflocculant plays an essential role in flocculating algal biomass.

The effects of different flocculation conditions including algal temperatures, algal pH, and metal ions on flocculation activity were investigated. Temperature is known to affect flocculation [23], therefore, the flocculation activity of bioflocculant under different algal temperatures were explored. The bioflocculant could exhibit high flocculation efficiency under 40 °C of algal temperature, compared to positive control. Fitzpatrick et al. have investigated the effects of temperature on floc formation, breakage and reformation, and confirmed that floc formation is slower and break-up is more reversible at lower temperatures, breakage is greater for higher temperatures [23], suggesting a weaker floc. For the bioflocculant FLC-hsn06, the floc could form under higher temperature, and floc formation maybe slow under lower temperature. When the algal temperature was low, the flocculation activity was limited. Therefore, the bioflocculant could play a higher role to harvest algal biomass under a relatively high temperature. Flocculation procedure is always influenced by algal initial pH through changing the surface charge distribution of algal cells, and there are reports evidenced that an increase on pH could cause the instantaneous flocs formation [24]. Algal pH causes enormous effects on flocculation activity of bioflocculant, and basic pH values are helpful to promote flocculation activity of the bioflocculant. This is different from the conclusion which determined by Ndikubwimana et al. The results from Ndikubwimana et al. showed that the flocculation efficiency of bacterial bioflocculant on microalgae *Desmodesmus* sp. F51 was dependent on the initial culture pH, the flocculation efficiency increased when the initial culture pH was changed from 7.2 to 3 [25]. Pérez et al. have reported that acid pH values (2–6) and basic pH values (8–12) have been tested to harvest *Skeletonema costatum* and *Chaetoceros gracilis* microalgae [26], which confirmed that the highest pH values (11, 11.5 and 12) caused higher flocculation activity than acid pH values, suggesting pH induced flocculation is an effective method for both species. Generally, the algal initial pH is weak alkaline, the bioflocculant FLC-hsn06 can be used to harvest algal biomass under basic pH values without changing algal initial pH values. Metal ions as coagulants can improve the flocculation activity of bioflocculant [16], some of the bioflocculants could flocculate algal cells only when metal ions were added together as coagulants [14, 15, 27]. Ca^2+^ ion could be selected as the most suitable coagulant to improve flocculation activity of the bioflocculant FLC-hsn06. Kim et al. used the bioflocculant produced by *Paenibacillus polymyxa* AM49 to Harvest *Scenedesmus* sp., and confirmed consecutive treatment with 8.5 mM CaCl_2_ and 0.2 mM FeCl_3_ as coagulants could improve the flocculation activity up to 95% [16]. Li et al. have reported that Ca^2+^ ion was best coagulant to improve the flocculation activity of the bioflocculant FLC-xn-1 [14]. Although metal ions could be added into algal culture as coagulants to improve flocculation activity, partial bioflocculants could exhibit high flocculation activity without adding metal ions. Wan et al. have confirmed that bioflocculant from *Solibacillus silvestris* could flocculate *Nannochloropsis oceanica* without adding any metal ions [17]. Yin et al. investigated the effect of metal ions on the flocculation activity of bioflocculant isolated from *Klebsiella* sp. ZZ-3 [28], the results showed that the bioflocculant could exhibit flocculation activity without adding metal ions, and the addition of Al^3+^, Fe^3+^ reduced the flocculation activity of the bioflocculant.

## Conclusions

The bioflocculant produced by an actinomycete *Streptomyces* sp. hsn06 with high flocculation activity on *Chlorella vulgaris* was investigated in this study. The bioflocculant was stable under wide ranges of temperature and pH, which was a nonprotein substance with moderate polarity as well as containing triple bond and cumulated double bonds. The bioflocculant was sensitive to algal temperatures and pH, and metal ions as coagulants were essential for flocculation activity. The bioflocculant could promote algal cells to form heavy floc to harvest algal cells. This bioflocculant possessed the potential to be widely used in harvesting *C. vulgaris* biomass.

## Additional files

Additional file 1: **Table S1.** The comparsion of bioflocculant from different microorganism (DOCX 20 kb)

Additional file 2: **Figure S2.** TLC analysis and HPLC analysis of the dichloromethane extract in bioflocculant. (JPG 127 kb)

Additional file 3: **Figure S1.** Component analysis of the bioflocculant. (JPG 76 kb)

## Abbreviations

*C. vulgaris*: *Chlorella vulgaris*
DMSO: Dimethyl sulfoxide
FITR: Fourier transform infrared
HPLC: High performance liquid chromatography
