# Supplementary material for: Flocculation characteristics of a bioflocculant produced by the actinomycete Streptomyces sp. hsn06 on microalgae biomass
Source: BMC Biotechnol. 2018 Sep 21;18:58. doi: 10.1186/s12896-018-0471-9 (PMC6151018; doi:10.1186/s12896-018-0471-9)
Supplement: Supplementary file 1 — Table S1. The comparsion of bioflocculant from different microorganism (DOCX 20 kb) [file 12896_2018_471_MOESM1_ESM.docx]

**Additional file**

**Figure Captions:**

**Fig. S1** Component analysis of the bioflocculant.

**Fig. S2** TLC analysis and HPLC analysis of the dichloromethane extract in bioflocculant.

**Table S1** The comparsion of bioflocculant from different microorganism

| Name | Type | Characteristics | Addition amount | Reference |
| --- | --- | --- | --- | --- |
| *Bacillus licheniformis* | Bacterium | carbohydrate and protein | Unknown | 11 |
| *Shinella albus* | Bacterium | Unknown | 30 mg/L | 14 |
| *Cobetia marina* | Bacterium | sugar | 20 mg/L | 15 |
| *Paenibacillus polymyxa* | Bacterium | Unknown | Unknown | 16 |
| Solibacillus silvestris | Bacterium | carbohydrate and protein | Unknown | 17 |
| *Bacillus amyloliquefaciens* | Bacterium | polysaccharides and protein | 1 mL | 21 |
| *Bacillus* licheniformis | Bacterium | γ-PGA | Unknown | 25 |
| *Klebsiella* | Bacterium | polysaccharides and protein | 1 mL | 28 |
| *Streptomyces* | Actinomycete | small molecule substance with containing triple bond and cumulated double bonds | 20 mg/L | This study |
