# Supplementary figures and images for: Flocculation characteristics of a bioflocculant produced by the actinomycete Streptomyces sp. hsn06 on microalgae biomass
Source: BMC Biotechnol. 2018 Sep 21;18:58. doi: 10.1186/s12896-018-0471-9 (PMC6151018; doi:10.1186/s12896-018-0471-9)

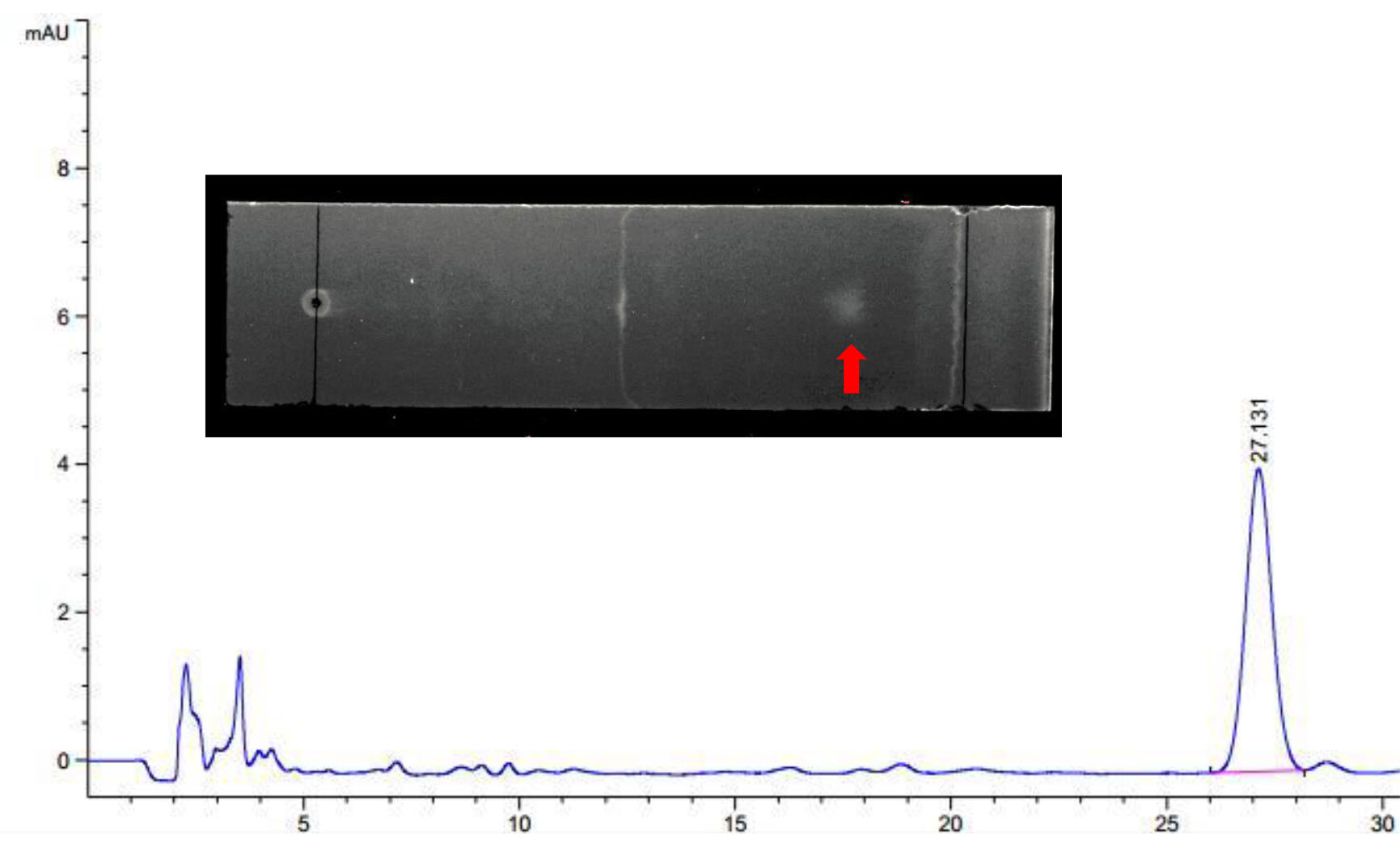

Supplement: Supplementary file 2 — Figure S2. TLC analysis and HPLC analysis of the dichloromethane extract in bioflocculant. (JPG 127 kb) [file 12896_2018_471_MOESM2_ESM.jpg]

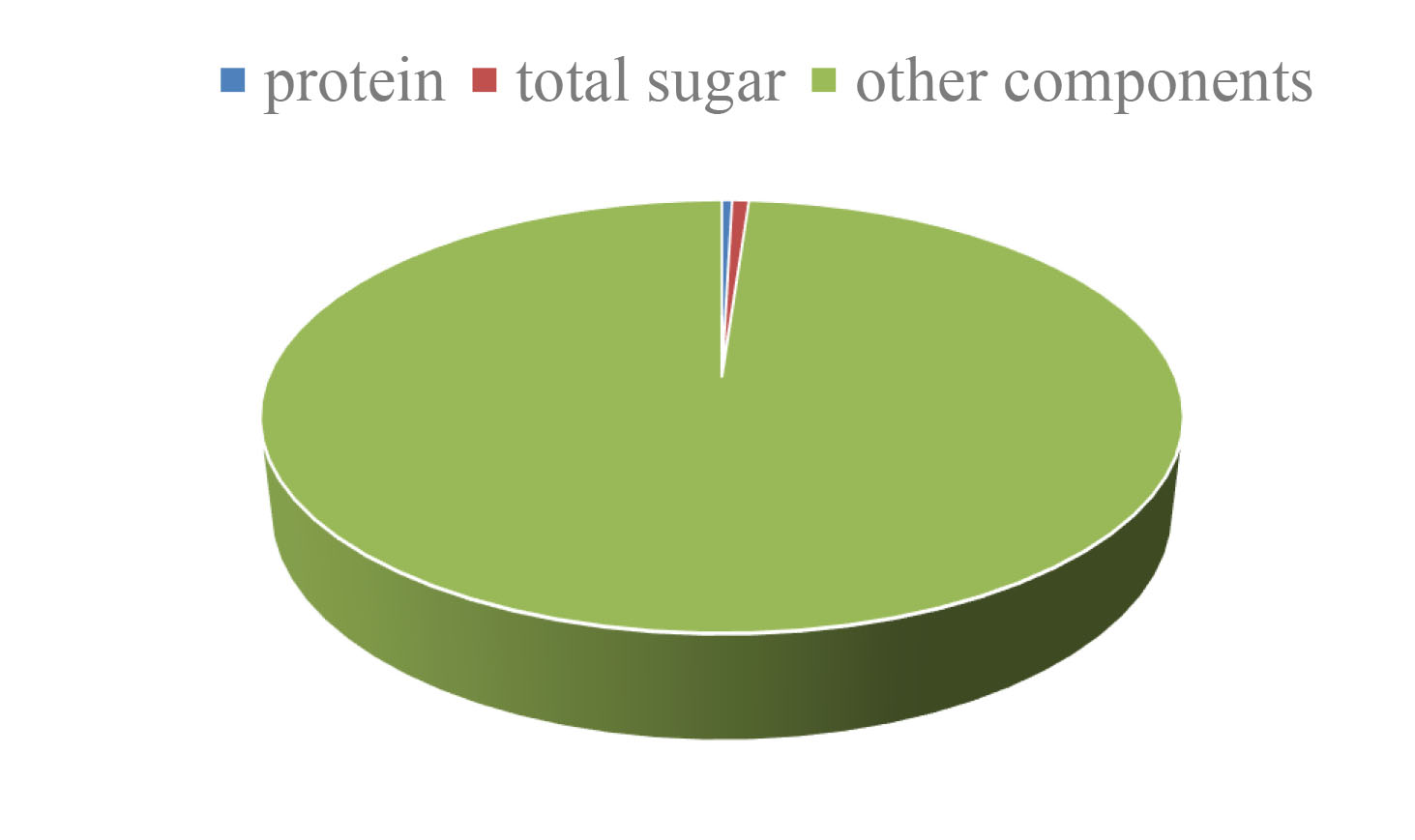

Supplement: Supplementary file 3 — Figure S1. Component analysis of the bioflocculant. (JPG 76 kb) [file 12896_2018_471_MOESM3_ESM.jpg]
